# Mortality After Partner’s Cancer Diagnosis or Death: A Population-based Prospective Cohort Study in Japan

**DOI:** 10.2188/jea.JE20240114

**Published:** 2025-03-05

**Authors:** Takeshi Makiuchi, Masako Kakizaki, Tomotaka Sobue, Tetsuhisa Kitamura, Hiroshi Yatsuya, Taiki Yamaji, Motoki Iwasaki, Manami Inoue, Shoichiro Tsugane, Norie Sawada

**Affiliations:** 1 Division of Environmental Medicine and Population Sciences, Graduate School of Medicine, Osaka University, Osaka, Japan; 2 Department of Medical Education, Nagoya City University School of Medicine, Aichi, Japan; 3 Department of Public Health and Health Systems, Nagoya University Graduate School of Medicine, Aichi, Japan; 4 Division of Epidemiology, National Cancer Center Institute for Cancer Control, Tokyo, Japan; 5 Division of Cohort Research, National Cancer Center Institute for Cancer Control, Tokyo, Japan; 6 Division of Prevention, National Cancer Center Institute for Cancer Control, Tokyo, Japan; 7 International University of Health and Welfare Graduate School of Public Health, Tokyo, Japan

**Keywords:** partner’s cancer diagnosis, partner’s death, mortality risk, suicide, prospective cohort study

## Abstract

**Background:**

The health statuses of closely connected individuals are interdependent. Little is known about mortality risk associated with partner’s cancer diagnosis and cause-specific mortality risk associated with partner’s death.

**Methods:**

Relative risks for all-cause and cause-specific mortality following a partner’s cancer diagnosis or death compared to the period when the partner is cancer-free and alive were investigated in the population-based prospective cohort study that enrolled 140,420 people at the age between 40–69 years in 1990–1994.

**Results:**

55,050 participants (27,665 men and 27,385 women) who were identified as married couples were followed-up for 1,073,746.1 (518,368.5 in men and 555,377.6 in women) person-years, during which 9,816 deaths were observed (7,217 in men and 2,599 in women). After a partner’s cancer diagnosis, the mortality rate ratio (MRR) of all-cause mortality was not increased among both men and women, while an increase of externally-caused MRR was observed. The suicide MRR significantly increased among men (MRR 2.90; 95% confidence interval, 1.70–4.93), and it persisted for more than 5 years. After a partner’s death, the MRRs of all-cause, cardiovascular disease (CVD), respiratory disease (RD), and externally-caused mortality significantly increased only among men. Stratified analysis by smoking status among men showed significantly increased MRRs of CVD and RD mortality among former/current smokers, but not among never-smokers.

**Conclusion:**

Partner’s cancer diagnosis did not increase all-cause mortality risk, but increased externally-caused mortality risk, especially suicide among men. The impact of partner’s death on mortality risk differed by the mortality causes and sex, and smoking affected some of cause-specific mortality risk.

## INTRODUCTION

The health statuses of closely connected people, such as married couples and families, are interdependent, and the deterioration in one person’s health has an impact on that of the others.^1^ Increased mortality risk after bereavement is well-known as the “widowhood effect,”^2^ and it has been suggested that the underlying mechanisms include psychological distress and changes in social ties, living arrangements, eating habits, and economic support.^2^^,^^3^ Increased risks of suicide and cardiovascular disease (CVD) mortality in people who were bereaved have been reported by several previous overseas studies^3^^–^^7^; however, previous studies that investigated the impact of bereavement on the other cause-specific mortality are limited. Increased risks of morality due to cancer; infection; respiratory disease (RD), including COPD; diabetes; kidney disease; and external causes, including accident and violence, were reported by one or a few previous studies in the United States, Scottland, and Norway.^2^^,^^8^^,^^9^ In Japan, only one study investigated risks of cause-specific mortality in people who were bereaved of spouses, in which increased risks of CVD, cancer, RD, and externally-caused mortality among men only were observed.^10^

One previous study demonstrated increased mortality associated with hospitalization of a spouse in people whose spouses were hospitalized.^11^ In this study, the impact of the illness of a spouse varied among diagnoses, and significant increase in mortality risk was associated with hospitalization for stroke, congestive heart failure, hip fracture, psychiatric disease, and dementia. The underlying mechanisms may include imposed stress and deprivation of social, emotional, economic, or other practical support.^11^^–^^13^ Cancer diagnosis is thought to have various impacts on the health of the cancer-diagnosed people’s spouses and family members, and previous studies have reported a decrease in quality of life (QoL), and an increase in the diagnosis of diseases, such as psychiatric illness, coronary heart disease, and stroke, in people whose spouses were diagnosed with cancer.^14^^–^^16^ However, the impact of cancer diagnosis on mortality among the cancer-diagnosed people’s spouses has been inconclusive because this has been studied by only a few studies and the results were inconsistent, and no study has investigated the cause-specific mortality associated with spousal cancer diagnosis.^11^^,^^17^^,^^18^

In summary, regarding mortality risk associated with partner’s health status change, evidence about cause-specific mortality risk associated with partner’s death is limited, especially in Japan, and impact of partner’s cancer diagnosis on mortality risk is still unclear. Therefore, we conducted this study to investigate risks of all-cause and cause-specific mortality associated with a partner’s cancer diagnosis and death in a large-scale population-based prospective cohort study conducted in Japan. We conducted stratified analysis by sex and smoking status because different effect of partner’s death by sex was suggested by previous studies,^10^^,^^19^ and the distribution of smoking status was remarkably different by sex in this study population.

## METHODS

### Study design and population

We used the data of Japan Public Health Center-based Prospective Study (JPHC Study), which investigates the lifestyle-related risk factors for non-communicable diseases. This study consists of two cohorts, and enrolled 140,420 individuals in total aged 40–69 years in 1990 and 1993–1994 for Cohorts I and II, respectively, and followed up for more than 20 years.^20^^,^^21^ The details including population, baseline questionnaire and follow up are described in eMaterial 1.

The JPHC study was approved by the Institutional Review Board of the National Cancer Center, Tokyo, Japan. This study was approved by the Ethical Review Board of Osaka University, Osaka, Japan.

### Baseline survey and paired data

A self-administered questionnaire asking about a variety of lifestyle habits was used as a baseline survey. The following factors were assessed as baseline characteristics: age, sex, smoking status (never smokers, former smokers, current smokers with <30 pack-years, current smokers with ≥30 pack-years, current smokers with unknown pack-years, and unknown), drinking status (non-drinkers, occasional drinkers, regular drinkers with ethanol consumption of <300 g/week, and ≥300 g/week, and unknown) body mass index (BMI; <23, 23–25, 25–27, or ≥27 kg/m^2^, and unknown) and employment status (employed, unemployed/housekeeper, and unknown). For participants who had already retired at baseline, employment status before retirement was used.

We used the available paired data about JPHC participants from the previous study that assessed the carcinogenic effect of spouse-related passive smoking exposure.^22^ Briefly, we identified 56,992 participants from 28,496 pairs (28,496 men and 28,496 women) of married couples by surname, address, sex, and age difference of <16 years. All female participants were never-smokers. We tested 644 pairs using residence registries to determine the accuracy of identification. The results showed that 604 pairs (93.8%) were married couples and six (0.9%) were relatives other than a spouse, while the relationship of 34 pairs (5.3%) could not be established.^22^

For the present study, the following participants were excluded from the identified paired participants: non-Japanese nationality (*n* = 1), previous history of cancer (*n* = 579), moving out of the study area before the follow-up start (*n* = 47), and missing of the date of follow-up end (*n* = 42). Additionally, 1,273 participants were excluded because of the following partner status: previous history of cancer, unknown cancer diagnosis date, cancer diagnosis information by death certificate only, or moving out of the study area before the start of follow-up.

### Follow-up

Study participants were followed up until the earliest of the followings: death date, date of moving out of the study area, or December 31, 2013 (the end of the study period). Participants moving out of the study area or lost to follow-up were censored on the last confirmed date of their presence in the study area. Of the 55,050 participants included in the data analysis, 9,816 died, 4,160 moved out, and 33 were lost to follow-up.

Cause-specific mortality was coded based on the International Classification of Diseases, 10th edition. The following major causes of death were used for analysis of cause-specific mortality: cancer (C00–C97); CVD (I00–I99); respiratory disease (RD) (J00–J99); gastrointestinal disease (GID) (K00–K93) and external causes (V01–Y98) which was further divided into suicide (X60–X84) and accident (V01–X59, X85–Y98).

### Statistical analysis

**Figure 1.**
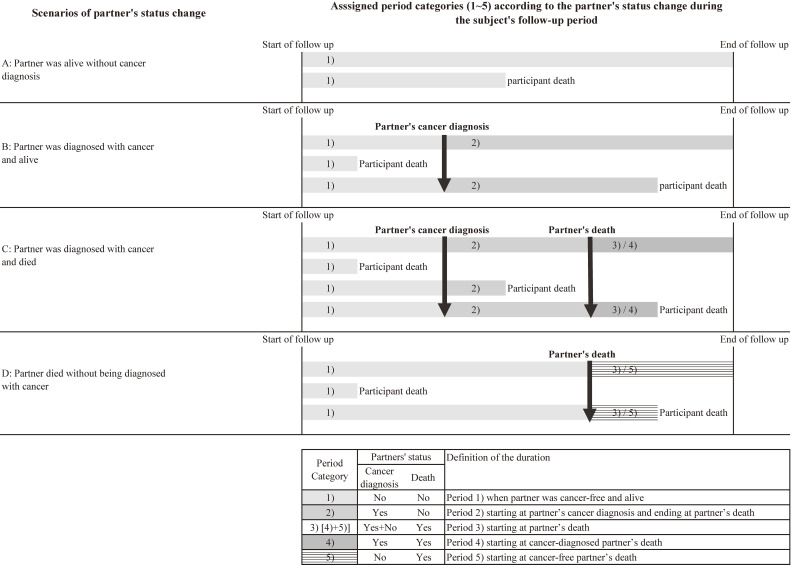
Definition of the study period categories by partner’s status

We classified the follow-up period into three categories: Period 1), when the partner was cancer-free and alive (control period); Period 2), starting at the partner’s cancer diagnosis and ending at the partner’s death; and Period 3), starting at partner’s death. Period 3) was further subdivided into Period 4), starting at cancer-diagnosed partner’s death; and Period 5), starting at cancer-free partner’s death (Figure 1). We calculated the mortality rate ratios (MRRs) and 95% confidence intervals (CIs) of all-cause and cause-specific mortality in periods 2), 3), 4), and 5) as compared to period 1) for all participants and each of men and women, using the Poisson regression model. We also calculated *P*-interaction to evaluate interaction of sex with the exposures. The multivariate model was adjusted for sex; attained age (5-year increments [eg, 40–44, 45–49, and 50–54]); study area (9 public health center areas); and some lifestyle, physical, or socioeconomic factors at baseline, including smoking status, drinking status, BMI, and employment status. We chose these factors because they were associated with the outcomes (ie, all-cause and/or some cause-specific mortality) and considered to be potentially associated with the exposures (ie, the spouse’ health status change including cancer diagnosis and death). Smoking, alcohol consumption, and BMI are reported for their associations with cancer and mortality risks,^23^^–^^27^ and spousal concordance in these factors were also reported by the previous studies,^28^^–^^30^ which suggests the possibility of their affecting spouse’s health status. Additionally, smoking affects the health of cohabiting spouse through passive smoking.^22^ Unemployment is reportedly associated with mortality risk,^31^ and it potentially affects the spousal health through the economic status shared within the family. To adjust for attained age, we divided the follow-up period into 5-year increments of the age attained (eg, 40, 45, and 50) and assigned the attained age to each split follow-up period. The analysis was further stratified by smoking status (never-smoker or former/current smoker) in men in order to explore interaction between the exposures and smoking, which may affect the results by sex because all female participants in this study were never-smokers.^22^ We calculated *P*-interaction to evaluate interaction between smoking status and the exposures. We conducted additional analysis by dividing periods 2) and 3) into the following 3 sub-periods: <1 year, 1–5 years, and ≥5 years after the start of the period. These sub-periods were determined referring to the previous studies.^17^^,^^18^ The MRRs of the sub-periods were calculated to understand the risk change over the course of time after a partner’s cancer diagnosis or death.

*P*-interaction was calculated by assigning nominal values to each variable and creating interaction term by multiplying nominal values for each variable. All *P* values reported were two-sided, and the significance level was set at *P* < 0.05. All statistical analyses were performed using Stata Version 17 software (Stata Corporation, College Station, TX, USA).

## RESULTS

The baseline characteristics of the participants (overall and separately for men and women) are shown in Table 1. A total of 55,050 participants (27,665 men and 27,385 women) were included in the analysis population. There were some differences of characteristics between men and women. Men tended to be older than women (average age: 53.8 vs 51.0 years). All women were never-smokers, while the proportion of never-smokers in men was 26.4%. Remarkably higher proportions in men than women were observed for regular drinker (66.8% vs 9.6%) and employed participant (96.3% vs 61.8%). The number of participants, duration of the follow-up period, and number of deaths by partner status are shown in Table 2. The eligible participants were followed-up for 1,073,746.1 person-years (518,368.5 person-years in men and 555,377.6 person-years in women), during which 9,816 deaths were observed (7,217 men and 2,599 women). The duration of the follow-up period differed according to the partner’s health status, and the cancer-free period (control period) was the longest, followed by the period after the partner’s death, and the period after the partner’s cancer diagnosis (before death). In the period after the partner’s cancer diagnosis (before death), the number of participants was higher in women, but the follow-up period/person was longer in men because, although both frequencies of cancer incidence and mortality were lower in women, the female-to-male ratio in mortality (1,949 vs 6,270) was lower than that in cancer incidence (2,357 vs 5,631).

**Table 1.** Demographic characteristics of participants at baseline

	All		Men		Women	
Number of participants	55,050		27,665		27,385	
Average age, years	52.4		53.8		51.0	
Smoking status, *n* (%)						
Never-smoker	34,685	(63.0)	7,300	(26.4)	27,385	(100.0)
Former smoker	6,671	(12.1)	6,671	(24.1)	0	(0.0)
Current smoker	13,619	(24.7)	13,619	(49.2)	0	(0.0)
Current smoker (<30 pack-year)	5,382	(9.8)	5,382	(19.5)	0	(0.0)
Current smoker (≥30 pack-year)	7,847	(14.3)	7,847	(28.4)	0	(0.0)
Current smoker (unknown pack-year)	390	(0.7)	390	(1.4)	0	(0.0)
Unknown	75	(0.1)	75	(0.3)	0	(0.0)
Drinking status, *n* (%)						
Non drinker	27,943	(50.8)	6,054	(21.9)	21,889	(79.9)
Occasional drinker	5,107	(9.3)	2,461	(8.9)	2,646	(9.7)
Regular drinker	21,109	(38.3)	18,477	(66.8)	2,632	(9.6)
Regular drinker (<300 g/week ethanol)	14,051	(25.5)	11,508	(41.6)	2,543	(9.3)
Regular drinker (≥300 g/week ethanol)	7,058	(12.8)	6,969	(25.2)	89	(0.3)
Unknown	891	(1.6)	673	(2.4)	218	(0.8)
Employment status, *n* (%)						
Employed	43,563	(79.1)	26,635	(96.3)	16,928	(61.8)
Unemployed/housekeeper	10,898	(19.8)	698	(2.5)	10,200	(37.2)
Unknown	589	(1.1)	332	(1.2)	257	(0.9)
BMI, kg/m^2^, *n* (%)						
<23	24,398	(44.3)	11,921	(43.1)	12,477	(45.6)
23–25	14,530	(26.4)	7,631	(27.6)	6,899	(25.2)
25–27	8,955	(16.3)	4,708	(17.0)	4,247	(15.5)
≥27	6,619	(12.0)	3,090	(11.2)	3,529	(12.9)
Unknown	548	(1.0)	315	(1.1)	233	(0.9)

BMI, body mass index.

**Table 2.** Number of participants, duration of follow-up period and number of mortality by partner’s status

Partner’s status	All		Period by partner’s status									

Death			No		No		Yes		Yes			

Cancer diagnosis			No		Yes		Yes+No		Yes		No	
All												
Person-years	1,073,746.1		971,044.1		38,478.0		64,223.9		30,419.5		33,804.4	
Number of participants	55,050		55,050		7,988		8,219		3,873		4,346	
Person-years/participants	19.5		17.6		4.8		7.8		7.9		7.8	
Mortality (all-cause)	9,816		8,356		459		1,001		441		560	
Cancer mortality, *n* (%)	4,123	(42.0)	3,607	(43.2)	184	(40.1)	332	(33.2)	143	(32.4)	189	(33.8)
CVD mortality, *n* (%)	2,457	(25.0)	2,059	(24.6)	111	(24.2)	287	(28.7)	128	(29.0)	159	(28.4)
RD mortality, *n* (%)	1,010	(10.3)	840	(10.1)	46	(10.0)	124	(12.4)	48	(10.9)	76	(13.6)
GID mortality, *n* (%)	356	(3.6)	296	(3.5)	14	(3.1)	46	(4.6)	30	(6.8)	16	(2.9)
Externally-caused mortality, *n* (%)	786	(8.0)	680	(8.1)	44	(9.6)	62	(6.2)	26	(5.9)	36	(6.4)
Suicide mortality, *n* (%)	291	(3.0)	253	(3.0)	21	(4.6)	17	(1.7)	6	(1.4)	11	(2.0)
Accidental mortality, *n* (%)	495	(5.0)	427	(5.1)	23	(5.0)	45	(4.5)	20	(4.5)	25	(4.5)
Others, *n* (%)	1,084	(11.0)	874	(10.5)	60	(13.1)	150	(15.0)	66	(15.0)	84	(15.0)
Men												
Person-years	518,368.5		491,396.2		13,301.9		13,670.4		6,585.2		7,085.2	
Number of participants	27,665		27,665		2,357		1,949		878		1,071	
Person-years/participants	18.7		17.8		5.6		7.0		7.5		6.6	
Mortality (all-cause)	7,217		6,454		266		497		213		284	
Cancer mortality, *n* (%)	3,040	(42.1)	2,767	(42.9)	110	(41.4)	163	(32.8)	74	(34.7)	89	(31.3)
CVD mortality, *n* (%)	1,798	(24.9)	1,587	(24.6)	69	(25.9)	142	(28.6)	59	(27.7)	83	(29.2)
RD mortality, *n* (%)	816	(11.3)	710	(11.0)	27	(10.2)	79	(15.9)	32	(15.0)	47	(16.5)
GID mortality, *n* (%)	267	(3.7)	241	(3.7)	8	(3.0)	18	(3.6)	13	(6.1)	5	(1.8)
Externally-caused mortality, *n* (%)	597	(8.3)	537	(8.3)	24	(9.0)	36	(7.2)	13	(6.1)	23	(8.1)
Suicide mortality, *n* (%)	218	(3.0)	193	(3.0)	15	(5.6)	10	(2.0)	3	(1.4)	7	(2.5)
Accidental mortality, *n* (%)	379	(5.3)	344	(5.3)	9	(3.4)	26	(5.2)	10	(4.7)	16	(5.6)
Others, *n* (%)	699	(9.7)	612	(9.5)	28	(10.5)	59	(11.9)	22	(10.3)	37	(13.0)
Women												
Person-years	555,377.6		479,647.9		25,176.1		50,553.5		23,834.3		26,719.3	
Number of paricipants	27,385		27,385		5,631		6,270		2,995		3,275	
Person-years/participants	20.3		17.5		4.5		8.1		8.0		8.2	
Mortality (all-cause)	2,599		1,902		193		504		228		276	
Cancer mortality, *n* (%)	1,083	(41.7)	840	(44.2)	74	(38.3)	169	(33.5)	69	(30.3)	100	(36.2)
CVD mortality, *n* (%)	659	(25.4)	472	(24.8)	42	(21.8)	145	(28.8)	69	(30.3)	76	(27.5)
RD mortality, *n* (%)	194	(7.5)	130	(6.8)	19	(9.8)	45	(8.9)	16	(7.0)	29	(10.5)
GID mortality, *n* (%)	89	(3.4)	55	(2.9)	6	(3.1)	28	(5.6)	17	(7.5)	11	(4.0)
Externally-caused mortality, *n* (%)	189	(7.3)	143	(7.5)	20	(10.4)	26	(5.2)	13	(5.7)	13	(4.7)
Suicide mortality, *n* (%)	73	(2.8)	60	(3.2)	6	(3.1)	7	(1.4)	3	(1.3)	4	(1.4)
Accidental mortality, *n* (%)	116	(4.5)	83	(4.4)	14	(7.3)	19	(3.8)	10	(4.4)	9	(3.3)
Others, *n* (%)	385	(14.8)	262	(13.8)	32	(16.6)	91	(18.1)	44	(19.3)	47	(17.0)

CVD, cardiovascular disease; GID, gastrointestinal disease; RD, respiratory disease.

The MRRs and 95% CIs of all-cause and cause-specific mortality, among all participants and separately for men and women, are shown in Table 3. After a partner’s cancer diagnosis, the risk trend differed between internally-caused and externally-caused mortality. The MRRs of all-cause and internally-caused mortality (including cancer, CVD, RD, and GID mortality) were not significantly increased among men and women, while the externally-caused MRR tended to increase among men and significantly increased among women (MRR 1.40; 95% CI, 0.93–2.12 and MRR 1.85; 95% CI, 1.14–3.01, respectively). The suicide MRR significantly increased among men but not among women (MRR 2.90; 95% CI, 1.70–4.93 and MRR 1.76; 95% CI, 0.74–4.17, respectively). The MRR of accidental mortality significantly increased among women (MRR 1.91; 95% CI, 1.07–3.44), but not among men, with a significant interaction with sex (*P*-interaction = 0.021).

**Table 3.** The MRRs and 95% CIs of all-cause and cause-specific mortality among all participants and each of men and women

Partner’s status	Period by partner’s status													

Death	No		No			Yes			Yes					

Cancer diagnosis	No		Yes			Yes+No			Yes			No		
				
	Mortality rate^a^	MRR^b^	Mortality rate^a^	MRR^b^	95% CI	Mortality rate^a^	MRR^b^	95% CI	Mortality rate^a^	MRR^b^	95% CI	Mortality rate^a^	MRR^b^	95% CI
**All participants**														
All-cause mortality	860.5	1.00^c^	1,192.9	1.04	0.94–1.14	1,558.6	1.22	1.13–1.30	1,449.7	1.18	1.07–1.30	1,656.6	1.25	1.14–1.36
Cancer mortality	371.5	1.00^c^	478.2	1.02	0.87–1.18	516.9	1.06	0.94–1.20	470.1	0.98	0.82–1.16	559.1	1.14	0.98–1.33
CVD mortality	212.0	1.00^c^	288.5	0.99	0.82–1.20	446.9	1.34	1.17–1.53	420.8	1.34	1.11–1.61	470.4	1.34	1.13–1.59
RD mortality	86.5	1.00^c^	119.5	0.91	0.68–1.23	193.1	1.21	0.99–1.47	157.8	1.08	0.80–1.46	224.8	1.30	1.02–1.66
GID mortality	30.5	1.00^c^	36.4	0.92	0.53–1.57	71.6	1.61	1.15–2.26	98.6	2.36	1.59–3.50	47.3	1.01	0.60–1.70
Externally-caused mortality	70.0	1.00^c^	114.4	1.60	1.17–2.18	96.5	1.41	1.07–1.86	85.5	1.29	0.86–1.93	106.5	1.52	1.07–2.15
Suicide mortality	26.1	1.00^c^	54.6	2.52	1.60–3.97	26.5	1.42	0.85–2.38	19.7	1.08	0.48–2.47	32.5	1.72	0.92–3.21
Accidental mortality	44.0	1.00^c^	59.8	1.20	0.78–1.83	70.1	1.40	1.01–1.94	65.7	1.36	0.86–2.16	74.0	1.44	0.94–2.19
**Men**														
All-cause mortality	1,313.4	1.00^c^	1,999.7	1.03	0.91–1.17	3,635.6	1.36	1.23–1.49	3,234.5	1.33	1.16–1.53	4,008.4	1.37	1.22–1.55
Cancer mortality	563.1	1.00^c^	826.9	1.03	0.85–1.25	1,192.4	1.16	0.99–1.36	1,123.7	1.16	0.92–1.46	1,256.1	1.16	0.94–1.44
CVD mortality	323.0	1.00^c^	518.7	1.08	0.85–1.37	1,038.7	1.53	1.28–1.83	895.9	1.48	1.14–1.92	1,171.5	1.57	1.25–1.97
RD mortality	144.5	1.00^c^	203.0	0.81	0.55–1.19	577.9	1.37	1.08–1.74	485.9	1.36	0.95–1.95	663.4	1.38	1.02–1.86
GID mortality	49.0	1.00^c^	60.1	0.84	0.42–1.71	131.7	1.34	0.82–2.20	197.4	2.26	1.28–3.99	70.6	0.65	0.26–1.59
Externally-caused mortality	109.3	1.00^c^	180.4	1.40	0.93–2.12	263.3	1.76	1.24–2.50	197.4	1.41	0.81–2.45	324.6	2.06	1.34–3.17
Suicide mortality	39.3	1.00^c^	112.8	2.90	1.70–4.93	73.2	1.86	0.97–3.56	45.6	1.18	0.38–3.72	98.8	2.47	1.14–5.36
Accidental mortality	70.0	1.00^c^	67.7	0.75	0.39–1.46	190.2	1.72	1.14–2.59	151.9	1.49	0.79–2.80	225.8	1.91	1.14–3.20
**Women**														
All-cause mortality	396.5	1.00^c^	766.6	1.04	0.90–1.21	997.0	1.07	0.97–1.19	956.6	1.04	0.90–1.20	1,033.0	1.11	0.97–1.26
*P*-interaction					0.929			0.001			0.015			0.026
Cancer mortality	175.1	1.00^c^	293.9	1.05	0.83–1.34	334.3	1.05	0.88–1.26	289.5	0.90	0.70–1.16	374.3	1.19	0.96–1.49
*P*-interaction					0.558			0.044			0.023			0.463
CVD mortality	98.4	1.00^c^	166.8	0.82	0.60–1.14	286.8	1.07	0.87–1.31	289.5	1.10	0.84–1.43	284.4	1.04	0.81–1.35
*P*-interaction					0.376			0.077			0.344			0.104
RD mortality	27.1	1.00^c^	75.5	1.14	0.70–1.85	89.0	0.96	0.67–1.38	67.1	0.74	0.43–1.26	108.5	1.16	0.76–1.76
*P*-interaction					0.276			0.074			0.054			0.414
GID mortality	11.5	1.00^c^	23.8	1.01	0.43–2.39	55.4	1.73	1.05–2.85	71.3	2.28	1.28–4.07	41.2	1.25	0.64–2.48
*P*-interaction					0.540			0.176			0.592			0.123
Externally-caused mortality	29.8	1.00^c^	79.4	1.85	1.14–3.01	51.4	0.98	0.62–1.54	54.5	1.05	0.58–1.91	48.7	0.91	0.50–1.65
*P*-interaction					0.245			0.153			0.791			0.093
Suicide mortality	12.5	1.00^c^	23.8	1.76	0.74–4.17	13.8	0.91	0.39–2.10	12.6	0.86	0.26–2.84	15.0	0.95	0.33–2.74
*P*-interaction					0.400			0.290			0.842			0.233
Accidental mortality	17.3	1.00^c^	55.6	1.91	1.07–3.44	37.6	1.02	0.60–1.75	42.0	1.15	0.58–2.28	33.7	0.91	0.44–1.86
*P*-interaction					0.021			0.327			0.880			0.225

CI, confidence interval; CVD, cardiovascular disease; GID, gastrointestinal disease; MRR, mortality rate ratio; RD, respiratory disease.

^a^Morality rate per 100,000 person-years.

^b^Mortality rate ratio adjusted for sex (for all participants only), attained age, smoking status, drinking status, employment status, and body mass index.

^c^Reference.

After a partner’s death, risk trends differed between genders. The MRR of all-cause mortality significantly increased among men (MRR 1.36; 95% CI, 1.23–1.49), but not among women, with a significant interaction with sex (*P*-interaction = 0.001). The MRRs significantly increased for CVD and RD, and slightly increased with borderline significance for cancer among men (MRR 1.53; 95% CI, 1.28–1.83, MRR 1.37; 95% CI, 1.08–1.74, and MRR 1.16; 95% CI, 0.99–1.36, respectively), but not among women, with borderline significance for sex interaction (*P*-interaction = 0.077, 0.074, and 0.044, respectively). The increase of GID MRR was significant among women (MRR 1.73; 95% CI, 1.05–2.85), but not among men. There was little difference in MRRs between periods after a cancer-diagnosed and cancer-free partner’s deaths for all-cause, cancer, CVD and RD mortality, while a trend of higher MRR after a cancer-diagnosed partner’s death was observed for GID mortality. For externally-caused, suicide and accidental mortality, the increased MRRs were observed among men, but not among women (MRR 1.76; 95% CI, 1.24–2.50, MRR 1.86; 95% CI, 0.97–3.56, and MRR 1.72; 95% CI, 1.14–2.59, respectively). For subperiod of the period after partner’s death, the MRRs after a cancer-free partner’s death significantly increased (MRR 2.06; 95% CI, 1.34–3.17, MRR 2.47; 95% CI, 1.14–5.36, and MRR 1.91; 95% CI, 1.14–3.20, respectively) and tended to be higher than those after a cancer-diagnosed partner’s death. They did not increase among women.

The MRRs and 95% CIs of all-cause and cause-specific mortality in men according to smoking status are shown in Table 4. After a partner’s cancer diagnosis, the observed trend of risks for all-cause, internally-caused and externally-caused mortality was similar between never-smokers and former/current smokers. The suicide MRR significantly increased among never-smokers and former/current smokers (MRR 3.71; 95% CI, 1.46–9.43 and MRR 2.62; 95% CI, 1.37–5.02, respectively), while the MRRs of the other death causes did not increase.

**Table 4.** The MRRs and 95% CIs of all-cause and cause-specific mortality among men stratified by smoking status

Partner’s status	Period by partner’s status													

Death	No		No			Yes			Yes					

Cancer diagnosis	No		Yes			Yes+No			Yes			No		
				
	Mortality rate^a^	MRR^b^	Mortality rate^a^	MRR^b^	95% CI	Mortality rate^a^	MRR^b^	95% CI	Mortality rate^a^	MRR^b^	95% CI	Mortality rate^a^	MRR^b^	95% CI
**Never-smokers**														
All-cause mortality	923.8	1.00^c^	1,460.5	1.10	0.83–1.46	2,438.2	1.27	1.02–1.57	2,116.0	1.29	0.93–1.79	2,700.6	1.25	0.95–1.64
Cancer mortality	345.4	1.00^c^	515.5	1.08	0.67–1.74	744.3	1.20	0.82–1.76	629.1	1.12	0.61–2.04	838.1	1.26	0.78–2.04
CVD mortality	259.2	1.00^c^	458.2	1.22	0.74–2.03	667.3	1.16	0.77–1.74	629.1	1.30	0.71–2.37	698.4	1.07	0.63–1.81
RD mortality	100.4	1.00^c^	114.5	0.68	0.25–1.84	179.7	0.60	0.28–1.30	114.4	0.50	0.12–2.04	232.8	0.65	0.26–1.62
GID mortality	23.2	1.00^c^	57.3	1.51	0.36–6.37	102.7	1.61	0.55–4.72	114.4	2.19	0.52–9.27	93.1	1.26	0.29–5.49
Externally-caused mortality	98.9	1.00^c^	200.5	1.69	0.79–3.64	308.0	2.24	1.21–4.13	171.6	1.36	0.43–4.29	419.1	2.89	1.43–5.86
Suicide mortality	37.5	1.00^c^	143.2	3.71	1.46–9.43	102.7	2.77	0.98–7.88	57.2	1.52	0.21–11.14	139.7	3.85	1.16–12.72
Accidental mortality	61.4	1.00^c^	57.3	0.73	0.18–2.97	205.3	2.02	0.95–4.30	114.4	1.27	0.31–5.21	279.4	2.55	1.07–6.08

**Former/Current smokers**														
All-cause mortality	1,455.6	1.00^c^	2,185.7	1.02	0.89–1.17	4,111.4	1.38	1.24–1.53	3,621.0	1.34	1.15–1.56	4,590.1	1.41	1.23–1.61
*P*-interaction					0.635			0.838			0.985			0.760
Cancer mortality	642.5	1.00^c^	939.6	1.03	0.83–1.27	1,367.0	1.14	0.96–1.37	1,290.2	1.15	0.89–1.48	1,442.0	1.14	0.90–1.45
*P*-interaction					0.920			0.706			0.962			0.591
CVD mortality	345.6	1.00^c^	531.1	1.04	0.78–1.37	1,192.3	1.66	1.37–2.02	998.9	1.56	1.16–2.08	1,381.1	1.75	1.36–2.25
*P*-interaction					0.532			0.217			0.745			0.174
RD mortality	160.9	1.00^c^	234.9	0.84	0.55–1.28	740.1	1.56	1.22–2.01	624.3	1.55	1.07–2.24	853.0	1.58	1.14–2.17
*P*-interaction					0.702			0.028			0.144			0.090
GID mortality	58.9	1.00^c^	61.3	0.74	0.33–1.68	143.9	1.28	0.73–2.23	228.9	2.29	1.24–4.25	60.9	0.48	0.15–1.52
*P*-interaction					0.303			0.319			0.733			0.146
Externally-caused mortality	113.2	1.00^c^	173.6	1.32	0.81–2.15	236.4	1.56	1.01–2.40	187.3	1.32	0.68–2.57	284.3	1.79	1.04–3.10
*P*-interaction					0.522			0.245			0.884			0.212
Suicide mortality	39.8	1.00^c^	102.1	2.62	1.37–5.02	61.7	1.55	0.67–3.59	41.6	1.10	0.27–4.47	81.2	1.97	0.71–5.44
*P*-interaction					0.490			0.408			0.773			0.449
Accidental mortality	73.4	1.00^c^	71.5	0.77	0.36–1.64	174.7	1.55	0.94–2.57	145.7	1.38	0.65–2.94	203.1	1.71	0.90–3.27
*P*-interaction					0.985			0.376			0.976			0.308

CI, confidence interval; CVD, cardiovascular disease; GID, gastrointestinal disease; MRR, mortality rate ratio; RD, respiratory disease.

^a^Morality rate per 100,000 person-years.

^b^Mortality rate ratio adjusted for attained age, smoking status (for former/current smoker only), drinking status, employment status, and body mass index.

^c^Reference.

After a partner’s death, the observed trend of risks differed by smoking status for CVD and RD mortality. The CVD and RD MRRs significantly increased among former/current smokers (MRR 1.66; 95% CI, 1.37–2.02 and MRR 1.56; 95% CI, 1.22–2.01, respectively), but not among never-smokers. Interaction with smoking status was significant for RD mortality, but not for CVD mortality (*P*-interaction = 0.028 and 0.217, respectively). The observed trend was similar between never-smokers and former-current smokers for the other death causes, with increased MRRs observed regardless of smoking status for all-cause, externally-caused, suicide and accidental mortality.

The MRRs and 95% CIs of all-cause and cause-specific mortality within 1 year, 1–5 years, and ≥5 years of the partner’s cancer diagnosis or death are shown in Table 5. After a partner’s cancer diagnosis, the suicide MRR tended to increase within a year, and significantly increased between 1–5 years and ≥5 years among men (MRR 2.56; 95% CI, 0.63–10.32, MRR 3.51; 95% CI, 1.65–7.49, and MRR 2.49; 95% CI, 1.10–5.65, respectively). For the other cause of mortality whose MRR significantly increased after a partner’s cancer diagnosis (shown in Table 3), the highest MRR was observed ≥5 years for externally-caused and accidental mortality among women (MRR 2.36; 95% CI, 1.25–4.45 and MRR 2.44; 95% CI, 1.15–5.17, respectively).

**Table 5.** The MRRs and 95% CIs of all-cause and cause-specific mortality within 1 year, 1–5 years and more than 5 years after partner’s cancer diagnosis or death

Partner’s status		Period by partner’s status													

Death		No				No					Yes				
Cancer diagnosis		No				Yes					Yes+No				
		
	Years after the start of the period	Person years	Cases	Morality rate^a^	MRR^b^	Person years	Cases	Mortality rate^a^	MRR^b^	95% CI	Person years	Cases	Mortality rate^a^	MRR^b^	95% CI
**Men**															

All-cause mortality	<1 year	491,396.2	6,454	1,313.4	1.00^c^	2,040.3	41	2,009.5	1.14	0.84–1.55	1,828.9	73	3,991.4	1.59	1.27–2.01
	1–5 years					5,085.9	93	1,828.6	1.01	0.82–1.24	5,508.1	171	3,104.5	1.21	1.04–1.41
	≥5 years					6,175.7	132	2,137.4	1.02	0.86–1.21	6,333.3	253	3,994.8	1.41	1.24–1.60

Cancer mortality	<1 year	491,396.2	2,767	563.1	1.00^c^	2,040.3	16	784.2	1.06	0.65–1.73	1,828.9	30	1,640.3	1.68	1.17–2.41
	1–5 years					5,085.9	34	668.5	0.88	0.63–1.23	5,508.1	53	962.2	0.96	0.73–1.27
	≥5 years					6,175.7	60	971.6	1.13	0.88–1.46	6,333.3	80	1,263.2	1.18	0.94–1.48

CVD mortality	<1 year	491,396.2	1,587	323.0	1.00^c^	2,040.3	10	490.1	1.12	0.60–2.09	1,828.9	23	1,257.6	2.01	1.33–3.04
	1–5 years					5,085.9	30	589.9	1.32	0.92–1.89	5,508.1	50	907.7	1.42	1.07–1.88
	≥5 years					6,175.7	29	469.6	0.89	0.62–1.29	6,333.3	69	1,089.5	1.50	1.17–1.91

RD mortality	<1 year	491,396.2	710	144.5	1.00^c^	2,040.3	7	343.1	1.55	0.73–3.26	1,828.9	10	546.8	1.43	0.76–2.67
	1–5 years					5,085.9	6	118.0	0.52	0.23–1.15	5,508.1	24	435.7	1.10	0.73–1.66
	≥5 years					6,175.7	14	226.7	0.81	0.48–1.37	6,333.3	45	710.5	1.56	1.15–2.12

GID mortality	<1 year	491,396.2	241	49.0	1.00^c^	2,040.3	0	0.0	0.00	––	1,828.9	1	54.7	0.59	0.08–4.25
	1–5 years					5,085.9	4	78.6	1.19	0.44–3.20	5,508.1	9	163.4	1.75	0.89–3.43
	≥5 years					6,175.7	4	64.8	0.83	0.31–2.23	6,333.3	8	126.3	1.22	0.60–2.49

Externally-caused mortality	<1 year	491,396.2	537	109.3	1.00^c^	2,040.3	3	147.0	1.20	0.39–3.74	1,828.9	4	218.7	1.51	0.56–4.06
	1–5 years					5,085.9	11	216.3	1.72	0.95–3.13	5,508.1	11	199.7	1.37	0.75–2.51
	≥5 years					6,175.7	10	161.9	1.21	0.65–2.28	6,333.3	21	331.6	2.15	1.38–3.37

Suicide mortality	<1 year	491,396.2	193	39.3	1.00^c^	2,040.3	2	98.0	2.56	0.63–10.32	1,828.9	2	109.4	2.79	0.69–11.30
	1–5 years					5,085.9	7	137.6	3.51	1.65–7.49	5,508.1	4	72.6	1.86	0.69–5.05
	≥5 years					6,175.7	6	97.2	2.49	1.10–5.65	6,333.3	4	63.2	1.58	0.58–4.31

Accidental mortality	<1 year	491,396.2	344	70.0	1.00^c^	2,040.3	1	49.0	0.58	0.08–4.13	1,828.9	2	109.4	1.03	0.25–4.13
	1–5 years					5,085.9	4	78.6	0.90	0.34–2.42	5,508.1	7	127.1	1.18	0.56–2.51
	≥5 years					6,175.7	4	64.8	0.69	0.26–1.86	6,333.3	17	268.4	2.35	1.43–3.88

**Women**															

All-cause mortality	<1 year	479,647.9	1,902	396.5	1.00^c^	4,654.9	29	623.0	1.03	0.71–1.49	5,959.4	57	956.5	1.20	0.92–1.56
	1–5 years					10,762.7	68	631.8	0.94	0.73–1.20	18,868.8	190	1,007.0	1.20	1.03–1.40
	≥5 years					9,758.4	96	983.8	1.14	0.93–1.40	25,725.3	257	999.0	0.97	0.85–1.12

Cancer mortality	<1 year	479,647.9	840	175.1	1.00^c^	4,654.9	13	279.3	1.13	0.65–1.96	5,959.4	19	318.8	1.11	0.70–1.76
	1–5 years					10,762.7	26	241.6	0.91	0.61–1.34	18,868.8	74	392.2	1.32	1.04–1.69
	≥5 years					9,758.4	35	358.7	1.16	0.82–1.64	25,725.3	76	295.4	0.87	0.68–1.11

CVD mortality	<1 year	479,647.9	472	98.4	1.00^c^	4,654.9	3	64.4	0.40	0.13–1.26	5,959.4	20	335.6	1.49	0.95–2.34
	1–5 years					10,762.7	14	130.1	0.72	0.42–1.22	18,868.8	52	275.6	1.16	0.86–1.55
	≥5 years					9,758.4	25	256.2	1.04	0.69–1.57	25,725.3	73	283.8	0.94	0.73–1.22

RD mortality	<1 year	479,647.9	130	27.1	1.00^c^	4,654.9	4	85.9	1.72	0.64–4.67	5,959.4	4	67.1	0.88	0.32–2.40
	1–5 years					10,762.7	6	55.7	0.95	0.42–2.17	18,868.8	12	63.6	0.79	0.43–1.43
	≥5 years					9,758.4	9	92.2	1.12	0.56–2.21	25,725.3	29	112.7	1.08	0.71–1.64

GID mortality	<1 year	479,647.9	55	11.5	1.00^c^	4,654.9	1	21.5	1.16	0.16–8.37	5,959.4	4	67.1	2.46	0.88–6.88
	1–5 years					10,762.7	2	18.6	0.88	0.21–3.62	18,868.8	16	84.8	2.95	1.64–5.30
	≥5 years					9,758.4	3	30.7	1.06	0.33–3.43	25,725.3	8	31.1	0.85	0.39–1.84

Externally-caused mortality	<1 year	479,647.9	143	29.8	1.00^c^	4,654.9	1	21.5	0.57	0.08–4.08	5,959.4	1	16.8	0.36	0.05–2.55
	1–5 years					10,762.7	8	74.3	1.85	0.90–3.80	18,868.8	12	63.6	1.31	0.71–2.41
	≥5 years					9,758.4	11	112.7	2.36	1.25–4.45	25,725.3	13	50.5	0.90	0.49–1.64

Suicide mortality	<1 year	479,647.9	60	12.5	1.00^c^	4,654.9	0	0.0	0.00	––	5,959.4	0	0.0	0.00	––
	1–5 years					10,762.7	3	27.9	2.07	0.64–6.70	18,868.8	6	31.8	2.14	0.89–5.14
	≥5 years					9,758.4	3	30.7	2.20	0.67–7.27	25,725.3	1	3.9	0.24	0.03–1.81

Accidental mortality	<1 year	479,647.9	83	17.3	1.00^c^	4,654.9	1	21.5	0.89	0.12–6.39	5,959.4	1	16.8	0.52	0.07–3.74
	1–5 years					10,762.7	5	46.5	1.75	0.70–4.35	18,868.8	6	31.8	0.95	0.41–2.22
	≥5 years					9,758.4	8	82.0	2.44	1.15–5.17	25,725.3	12	46.6	1.18	0.62–2.25

CI, confidence interval; CVD, cardiovascular disease; GID, gastrointestinal disease; MRR, mortality rate ratio; RD, respiratory disease.

^a^Morality rate per 100,000 person-years.

^b^Mortality rate ratio adjusted for attained age, smoking status, drinking status, employment status, and body mass index.

^c^Reference.

After a partner’s death, the MRRs of all-cause and CVD mortality significantly increased in all three periods among men. For causes of mortality whose MRR significantly increased after a partner’s death (shown in Table 3), the highest MRR was observed within a year for all-cause and CVD mortality among men (MRR 1.59; 95% CI, 1.27–2.01 and MRR 2.01; 95% CI, 1.33–3.04, respectively), between 1–5 years for GID mortality among women (MRR 2.95; 95% CI, 1.64–5.30), and ≥5 years for externally-caused and accidental mortality among men (MRR 2.15; 95% CI, 1.38–3.37 and MRR 2.35; 95% CI, 1.43–3.88, respectively).

## DISCUSSION

In a large-scale, population-based prospective cohort study in Japan, we investigated the risks of all-cause and cause-specific mortality in different periods after a partner’s cancer diagnosis and death, compared to the period when the partner was cancer-free and alive. The results showed a different risk trend according to the cause of mortality, sex, and smoking status in men.

The present study did not show an increased risk of all-cause mortality after a partner’s cancer diagnosis, irrespective of sex or the early or late period after cancer diagnosis. Previous studies have been inconclusive about the association between mortality risk and spousal cancer diagnosis. In a previous cohort study in the United States, mortality risk was not increased after spousal hospitalization due to cancer diagnosis.^11^ A significant but slight increase in mortality risk among men was shown in a study conducted in Denmark (HR 1.03; 95% CI, 1.01–1.05).^17^ In a Japanese study, the risk of mortality was not significantly increased throughout the whole period following the partner’s cancer diagnosis, but a significant increase was observed only during the early period (within 1 year after the partner’s cancer diagnosis).^18^ The results from the present study and these previous studies suggest that the effect of a partner’s cancer diagnosis on all-cause mortality risk may be null or marginal. The underlying mechanism for the suggested effect is unclear. Some previous studies reported beneficial effect of caregiving on health; therefore, this may offset the mental and physical burden from partner’s cancer diagnosis in total.^32^^–^^34^

The present study showed a remarkable increase of suicide risk after a partner’s cancer diagnosis among men (MRR 2.90; 95% CI, 1.70–4.93). Previous studies reported that cancer diagnosis might impair quality of life and increase economic burden, psychological distress, and psychiatric disease among cancer patients’ partners or caregivers.^14^^,^^15^^,^^35^^,^^36^ Since mental disorders are associated with suicide risk, impaired mental health caused by a partner’s cancer diagnosis might have caused the increased suicide risk observed in the present study.^37^ Furthermore, this study showed that suicide risk among men continued to be high even more than 5 years after the partner’s cancer diagnosis, demonstrating the seriousness of the accumulated burden and the necessity of long-term support for spousal caregivers for patients with cancer.

An increased risk of suicide mortality was observed after cancer-free partner’s death among men, but not among women. This result is consistent with the previous study,^4^ and the increased risk is considered caused by serious mental condition after bereavement.^5^ On the other hand, an increased risk was not observed after a cancer-diagnosed partner’s death, although it was observed after partner’s cancer diagnosis. This may be due to a relief from caregiving burden. Additionally, the impact of bereavement from a cancer-diagnosed partner on mental condition may be smaller than that from a cancer-free partner, because a previous study reported that unexpected bereavement has a greater mortality impact than bereavement preceded by chronic disease.^38^

The present study showed that the risks of all-cause, CVD, RD, and externally caused mortality increased after a partner’s death in men but not in women. This observed difference by sex was consistent with a previous study in Japan.^10^ Additionally, a previous meta-analysis demonstrated that men were more affected by spousal death than women.^19^ However, in the stratified analysis by smoking status, the risks of all-cause, CVD, and RD mortality were significantly increased with greater effect size among male former/current smokers than among all men, whereas, the risk of all-cause mortality was increased with borderline significance, and the risks of CVD and RD were not significantly increased among male never-smokers. The observed results for externally caused mortality were similar between former/current smokers and never-smokers. These findings from the stratified analysis by smoking status and the comparison between women (all never-smokers) and male never-smokers suggest that smoking may contribute to some of the observed sex-related differences in the increased risks of internally caused mortality, such as CVD and RD. Smoking is closely associated with onset of and death due to CVD and RD.^39^^,^^40^ The impact of bereavement on health is thought to be mediated by various behavioral changes and biological effects, including immune/inflammatory changes and genetic/epigenetic changes induced by psychological distress.^6^^,^^41^ These effects might enhance the smoking effect to accelerate CVD and RD onset and mortality, resulting in the observed sex-related differences in the present study. To our knowledge, this is the first study to investigate the mortality risk associated with bereavement stratified by smoking status. The result that the risk of CVD mortality increased was only observed among widowed men but not among widowed women in a previous study where the proportion of smokers was higher than 50% among men and less than 10% among women does not contradict this hypothesis.^10^ The limitation regarding this point is that this study did not include women with smoking experience.

The strengths of this study include its prospective design, long follow-up period, availability of information on the cause of death, and large sample size. These factors enabled the evaluation of detailed individual cause-specific mortality and stratified analysis by sex and smoking status. To our knowledge, this is the first study to investigate multiple high-frequent cause-specific mortality risks associated with cancer diagnosis in a partner. Additionally, information about the timing of cancer diagnosis and death during follow-up was available in the study, which enabled the investigation of risk changes over time (ie, within 1 year, 1–5 years, and >5 years) following the partner’s cancer diagnosis and death.

The present study has several limitations. First, despite the large-scale design and long follow-up period, the sample size was not sufficient to analyze less frequent mortality, such as suicide, in detail. Second, there may be some misclassification of married couple identification, and some may not be actual married couples but relatives living together, although high accuracy in identifying married couple was confirmed in the previous study.^22^

In conclusion, partner’s cancer diagnosis did not increase all-cause mortality risk, but increased externally-caused mortality risk, especially suicide among men. The impact of partner’s death on mortality risk differed by the mortality causes and sex, and smoking affected some of cause-specific mortality risk. These results clarified the areas to be cared for in people whose partners were diagnosed with cancer or died, which will help the future discussion about how to support them.

## SUPPLEMENTARY MATERIAL

eMaterial 1. Study design of the JPHC study
