# Supplementary material for: Mortality After Partner’s Cancer Diagnosis or Death: A Population-based Prospective Cohort Study in Japan
Source: J Epidemiol. 2025 Mar 5;35(3):118–28. doi: 10.2188/jea.JE20240114 (PMC11821380; doi:10.2188/jea.JE20240114)
Supplement: Supplementary file 1 [file je-35-118-s001.pdf]

eMaterial 1. Study design of the JPHC study

The JPHC study is a population-based cohort study that comprise two cohorts and enrolled 140,420 individuals. Cohort I comprises five public health center (PHC) areas and started in 1990, and cohort II comprises six PHC areas and started in 1993. All the residents at the age between 40–59 years as of 31-Dec-1989 for cohort I, or 40–69 years as of 31-Dec-1992 for cohort II, living in the city and town in each PHC areas were selected as participants.[1] We excluded data from the Tokyo and Osaka areas because the cancer incidence data was not available in Tokyo, and participants in Osaka were not fully population-based since it included health check-up examinees and randomly sampled individuals. Details of the JPHC study, including the location of the study areas and number of study subjects, have been described previously.[1]

A self-administered questionnaire was used to obtain information on the participant's sociodemographic characteristics and personal medical history, including smoking and lifestyle habits. The questionnaire was distributed as a baseline survey in 1990 and 1993–1994 for Cohorts I and II, respectively, and 113,461 participants responded (approximately 81% response rate).

The Basic Resident Registration Act in Japan mandates residency registration. Follow-up was conducted using information about residential status and survival collected from the

residency registration of each municipality in the study area.[2]

Death certificates collected from local PHCs were used to confirm the cause of death, with permission from the Ministry of Health, Labour and Welfare. Cause-specific mortality was assessed based on the International Classification of Diseases, 10th edition (ICD-10).

The cancer incidence was identified through active patient notification by major local hospitals in the study area and by data linkage with population-based cancer registries. Death certificates were used as supplementary information sources to obtain complete information on cancer incidence. Patients with cancer were coded according to the International Classification of Diseases for Oncology, Third Edition (ICD-O-3).

REFERENCES

1. Tsugane S, Sawada N. The JPHC study: Design and some findings on the typical Japanese diet. *Jpn J Clin Oncol*. 2014;44:777-782.

2. Watanabe S, Tsugane S, Sobue T, Konishi M, and Baba S. Study design and organization of the JPHC study. Japan Public Health Center-based Prospective Study on Cancer and Cardiovascular Diseases. *J Epidemiol*. 2001;11: S3-7.
